# IGF1R as a Potential Pharmacological Target in Allergic Asthma

**DOI:** 10.3390/biomedicines9080912

**Published:** 2021-07-29

**Authors:** Elvira Alfaro-Arnedo, Icíar P. López, Sergio Piñeiro-Hermida, Álvaro C. Ucero, Francisco J. González-Barcala, Francisco J. Salgado, José G. Pichel

**Affiliations:** 1 Lung Cancer and Respiratory Diseases Unit, Center for Biomedical Research of La Rioja (CIBIR), Fundación Rioja Salud, 26006 Logroño, Spain; ealfaro@riojasalud.es (E.A.-A.); iplgarcia@riojasalud.es (I.P.L.); 2 Telomeres and Telomerase Group, Molecular Oncology Program, Spanish National Cancer Centre (CNIO), 28029 Madrid, Spain; spineiro@cnio.es; 3 Thoracic Oncology, Research Institute Hospital 12 de Octubre, 28041 Madrid, Spain; acuceroh@ucm.es; 4 Department of Physiology, Faculty of Medicine, Complutense University, 28040 Madrid, Spain; 5 Department of Respiratory Medicine, University Hospital of Santiago de Compostela (CHUS), 15706 Santiago de Compostela, Spain; francisco.javier.gonzalez.barcala@sergas.es; 6 Health Research Institute of Santiago de Compostela (FIDIS), 15706 Santiago de Compostela, Spain; 7 Spanish Biomedical Research Networking Centre-CIBERES, 15706 Santiago de Compostela, Spain; 8 Department of Biochemistry and Molecular Biology, Faculty of Biology-Biological Research Centre (CIBUS), Universidad de Santiago de Compostela, 15706 Santiago de Compostela, Spain; franciscojavier.salgado@usc.es

**Keywords:** asthma, allergy, house dust mite, IGF1R, NVP-ADW742, pharmacological blockade

## Abstract

Background: Asthma is a chronic lung disease characterized by reversible airflow obstruction, airway hyperresponsiveness (AHR), mucus overproduction and inflammation. Although Insulin-like growth factor 1 receptor (IGF1R) was found to be involved in asthma, its pharmacological inhibition has not previously been investigated in this pathology. We aimed to determine if therapeutic targeting of IGF1R ameliorates allergic airway inflammation in a murine model of asthma. Methods: C57BL/6J mice were challenged by house dust mite (HDM) extract or PBS for four weeks and therapeutically treated with the IGF1R tyrosine kinase inhibitor (TKI) NVP-ADW742 (NVP) once allergic phenotype was established. Results: Lungs of HDM-challenged mice exhibited a significant increase in phospho-IGF1R levels, incremented AHR, airway remodeling, eosinophilia and allergic inflammation, as well as altered pulmonary surfactant expression, all of being these parameters counteracted by NVP treatment. HDM-challenged lungs also displayed augmented expression of the IGF1R signaling mediator p-ERK1/2, which was greatly reduced upon treatment with NVP. Conclusions: Our results demonstrate that IGF1R could be considered a potential pharmacological target in murine HDM-induced asthma and a candidate biomarker in allergic asthma.

## 1. Introduction

Asthma is a chronic inflammatory disease characterized by reversible airflow obstruction, airway hyperresponsiveness (AHR) and inflammation [1]. The house dust mite (HDM) is ubiquitous in human habitats and a significant factor underlying allergic asthma since 65 to 130 million people worldwide are sensitive to HDM [2,3] Most asthmatics are well controlled on inhaled corticosteroids, but some patients, including those with eosinophilic asthma, continue to experience symptoms and exacerbations, with no effective treatments [4]. In this sense, the identification of asthma phenotypes, as well as underling Th2-high (eosinophilic) or Th2-low (noeosinophilic) endotypes, represent a key point for the development of novel therapeutic strategies [5].

The insulin-like growth factor 1 receptor (IGF1R) is a ubiquitously expressed membrane-bound tyrosine kinase receptor that recognizes its two major ligands, IGF1 and IGF2. IGF activity is modulated by six high-affinity IGF binding proteins (IGFBPs) that control multiple essential cellular functions [6]. IGF activity is highly relevant in several chronic lung pathologies with an inflammatory component [7,8,9]. Accordingly, IGF1R was recently identified as a novel outcome biomarker in critical COVID-19 patients to predict mortality [10], but it has not been evaluated in asthmatics.

Specifically, IGF1 signaling has been implicated in activation of different aspects of the asthmatic response and IGFBP3 was suggested to be involved in allergic airway inflammation [7]. On the other hand, the serum biomarker IGF-ALS (IGF Binding Protein Acid Labile Subunit) was recently reported to be capable of differentiating moderate-severe allergic from non-allergic asthma [11]. In addition, IGF1R was found to be upregulated in eosinophils from bronchoalveolar lavage of mild asthmatic patients [12]. In mice, IGF1 mediates allergic airway inflammation, and IGFBP3 was shown to block the effects of asthma [13,14]. Notably, we have reported that IGF1R plays a relevant role in initiation of the inflammatory response and that *Igf1r*-gene targeting in mice attenuates allergic airway inflammation [15,16,17].

Anti-IGF1R therapies, including small tyrosine kinase inhibitors (TKIs), continue to be valid targets for patients with cancer [18,19]. In this sense, the TKI NVP-ADW742 (NVP) was reported to suppress multiple myeloma tumor growth [20]. However, the IGF1R inhibitor NVP has still not been investigated in asthma. Here, we aimed to determine if therapeutic targeting of IGF1R ameliorates allergic airway inflammation in a murine model of asthma. For this purpose, C57BL/6J mice were challenged with HDM extract and therapeutically treated with NVP.

## 2. Materials and Methods

### 2.1. HDM Sensitization Protocol and Therapeutic Inhibition of IGF1R

**Figure 1:**
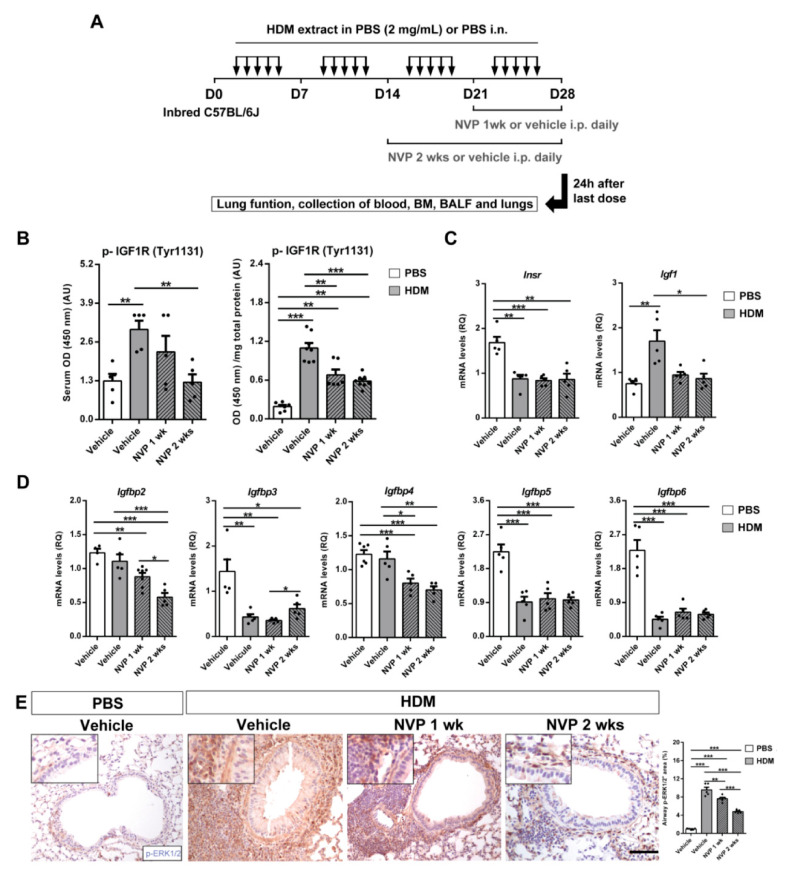
Protocol for HDM exposure and treatment with the IGF1R inhibitor NVP, as well as p-IGF1R and IGF system gene expression levels in the lung. (**A**) Mice were challenged by intranasal (i.n.) administration of HDM extract in phosphate buffer saline (PBS) or equal volume of vehicle, five days a week for four weeks. Mice also received intraperitoneal (i.p.) injections of the IGF1R inhibitor NVP or equal volume of the vehicle (2% DMSO) twice daily during the last one (NVP 1 week) or two weeks (NVP 2 weeks) of the HDM protocol. Lung function assessment and collection of blood, bone marrow (BM), BALF and lungs were performed 24h after the last exposure on day (**D**) 28. (**B**) p-IGF1R protein levels in both serum and lung homogenates from HDM-challenged mice treated with NVP vs. controls (*n* = 6–8 mice per group). (**C**,**D**) Lung tissue mRNA expression of IGF system-related genes *Insr*, *Igf1* (**C**), and *Igfbp2, Igfbp3, Igfbp4, Igfbp5, Igfbp6* (**D**) normalized to 18S expression in HDM-challenged mice treated with NVP vs. controls (*n* = 5 mice per group). (**E**) Representative immunostains of proximal airways for p-ERK1/2 (p-42/44) (brown), and quantification of p-ERK1/2^+^ area (%) in lung sections from HDM-challenged mice treated with NVP vs. controls (*n* = 5 mice per group; scale bar: 50 µm). Insets illustrate *p-ERK1/2* expression in smooth muscle cells and peribronchiolar areas. Data are expressed as mean ± SEM. * *p* < 0.05; ** *p* < 0.01; *** *p* < 0.001 (Mann–Whitney U test or Student´s *t*-test for comparing two groups, and Kruskal–Wallis test or ANOVA multiple comparison test for grouped or multivariate analysis).

Eight-week-old C57BL/6J mice were intranasally (i.n.) challenged with 40 μg of house dust mite (HDM) extract or PBS for four weeks [15]. Mice were also intraperitoneally (i.p.) injected with 200 μL of IGF1R tyrosine kinase inhibitor (TKI) NVP-ADW742 or vehicle (DMSO) twice a day during the last one or two weeks of the HDM protocol (Figure 1A). The concentration and dose-response of NVP were previously assessed by Mitsiades et al. [20] and Cintron-Colon et al. [21], respectively. For additional details, see Supplementary Materials.

### 2.2. In Vivo Assessment of Lung Function

Mice were anesthetized, intubated with a 24-gauge catheter and intravenously injected with 1 mg/kg of methacholine (MCh). Lung function was assessed in a plethysmograph to determine LR (lung resistance) and Cdyn (dynamic compliance). For additional details, see Supplementary Materials.

### 2.3. Sample Collection and Preparation

Animals were euthanized using 10 μL/g of ketamine-xylazine. Blood was then collected and lungs were lavaged with PBS. Right lung lobes were dissected and snap frozen for quantitative PCR (qPCR) and ELISA analyses, and the left lung lobe was harvested for histopathological evaluation or immunohistochemistry. Femurs were dissected to isolate bone marrow. For additional details, see Supplementary Materials.

### 2.4. Histopathological and Immunostaining Analysis

Hematoxylin and eosin (H&E) staining was performed for the quantification of inflammation and to assess airway thickness. Periodic acid-Schiff (PAS) and Masson’s trichrome staining served to evaluate the number of mucus-producing cells and collagen deposition. p-ERK1/2 (p-42/44), CD45 and SMA antibodies were used to evaluate airway p-ERK1/2+ and CD45+ areas, and smooth muscle thickness. SFTPC antibody was used to determine the number of SFTPC+ cells in the lung. SOX2, SCGB1A1 and MUC5AC antibodies served to quantify the degree of bronchial differentiation and for the assessment of goblet cell hyperplasia, respectively. For additional details, see Supplementary Materials.

### 2.5. RNA Isolation, Reverse Transcription and qPCR 

Inferior right lung lobes were homogenized in TRIzol, and then RNA was isolated and reverse-transcribed to cDNA. cDNA samples were amplified by qPCR for each primer pair assayed (Table S1). Results were normalized using the 18S rRNA gene (Rn18s). For additional details, see Supplementary Materials.

### 2.6. Mouse ELISAS 

Serum total IgE, IL13 and p-IGF1R levels were assessed with mouse ELISA kits. Superior right lung lobes were homogenized in RIPA Buffer. IL13, IL33, CCL11 and p-IGF1R levels were evaluated in homogenized lung tissue lysates using mouse ELISA kits, and normalized to total lung protein levels. For additional details, see Supplementary Materials.

### 2.7. Statistics 

Following a Shapiro–Wilk normality test, the statistical significance was determined using the Mann–Whitney U test or Student’s *t*-test for comparing 2 groups and the Kruskal–Wallis test or ANOVA multiple comparison test for grouped or multivariate analysis. Statistical analyses were carried out using SPSS Statistics Software v21 for Windows (IBM, Armonk, NY, USA). For all analysis, a *p* value < 0.05 was considered statistically significant. 

## 3. Results

### 3.1. Efficient Depletion of IGF1R and IGF System Gene Expression upon HDM Exposure and Pharmacological Blockade of IGF1R 

C57BL/6J mice were challenged with HDM extract and therapeutically treated with NVP (Figure 1A). NVP administration did not induce significant changes in body weight (Figure S1). Noteworthy, treatment with the vehicle of NVP (DMSO) did not induce inflammation in lungs of inbred C57BL/6J mice (Figure S2). In accordance, several drugs were previously reported to be dissolved in up to 5% DMSO for their use in preclinical mouse models [22,23].

IGF1R inhibition and assessment of IGF system gene expression were performed on lung extracts of HDM-challenged mice treated with NVP vs. controls (HDM + vehicle and PBS + vehicle) (Figure 1B–D). Phospho(p)-IGF1R levels quantified by ELISA were greatly increased in HDM control mice both in serum and lung homogenates. This increment was significantly reduced in NVP-treated mice particularly NVP after 2 weeks of treatment (NVP 2 weeks) (Figure 1B). mRNA levels of insulin receptor (*Insr*) were significantly decreased in all HDM-challenged groups. In addition, HDM treatment increased *Igf1* mRNA levels that were reverted by NVP treatment (Figure 1C). Regarding mRNA expression of IGFBP markers, *Igfbp2* and *Igfbp4* were found to be significantly reduced in NVP-treated mice and *Igfbp3*, *Igfbp5* and *Igfbp6* levels showed a significant depletion upon HDM exposure. Specifically, *Igfbp3* levels were found slightly increased in the NVP 2 weeks group (Figure 1D). In addition, lung tissue p-ERK1/2 (p42/44) expression evaluated by immunohistochemistry was highly augmented in HDM controls while this increment was significantly reduced in NVP-treated mice particularly after 2 weeks of treatment. Interestingly, expression pattern of p-ERK1/2 was specifically noticed in peribronchiolar smooth muscle cells and inflammatory areas (Figure 1E). 

### 3.2. Therapeutic Inhibition of IGF1R during HDM Exposure Attenuates Peripheral Blood and Bone Marrow Eosinophilia and the Increase in Serum IL13 

**Figure 2:**
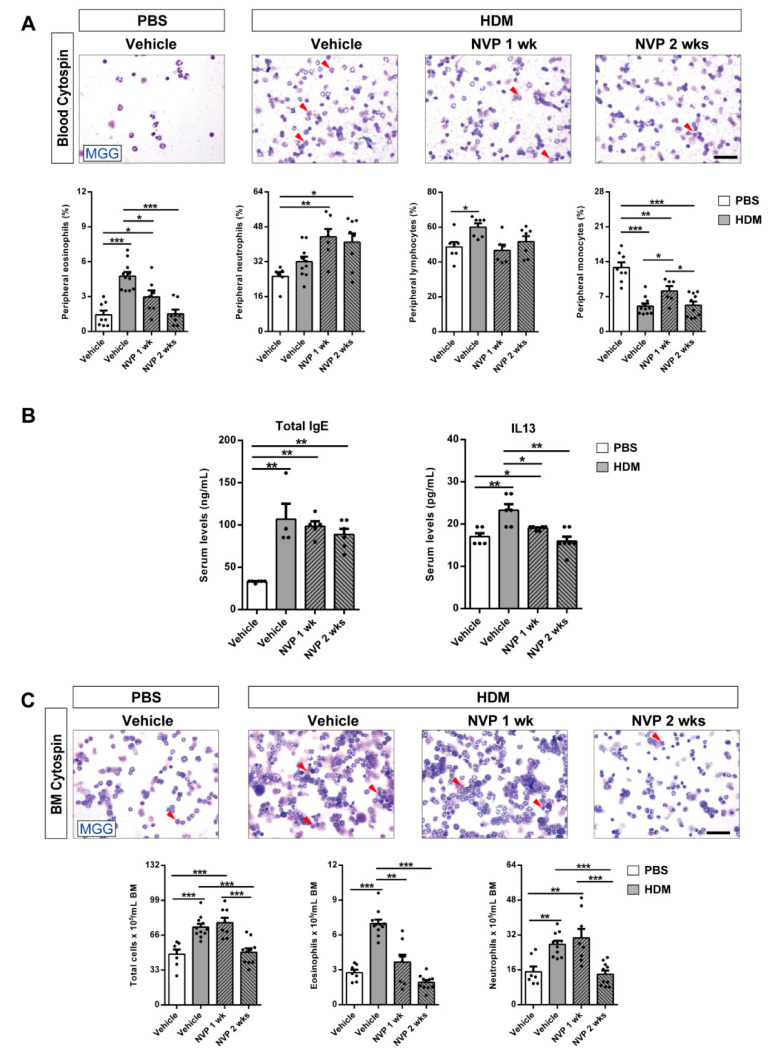
Pharmacological blockade of IGF1R depletes eosinophil presence in peripheral blood and bone marrow and attenuates the increase in serum IL13 levels after HDM exposure. (**A**,**C**) Representative images showing May-Grünwald/Giemsa (MGG) stained peripheral blood and bone marrow cytospin preparations (red arrowheads indicate eosinophils), and differential cell counts for eosinophils, neutrophils, lymphocytes and monocytes in peripheral blood (**A**), and total cells, eosinophils and neutrophils in bone marrow (**C**) from HDM-challenged mice treated with NVP vs. controls (*n* = 7–10 mice per group; scale bars: 50 µm). (**B**) Total serum IgE and IL13 levels from HDM-challenged mice treated with NVP vs. controls (*n* = 5–7 mice per group). Data are expressed as mean ± SEM. * *p* < 0.05; ** *p* < 0.01; *** *p* < 0.001 (Mann–Whitney U test or Student´s *t*-test for comparing two groups and Kruskal–Wallis test or ANOVA multiple comparison test for grouped or multivariate analysis).

We first assessed peripheral blood cellularity and serum IgE and IL13 levels in HDM-challenged mice treated with NVP vs. controls (Figure 2A,B). The proportion of eosinophils exhibited a marked increase in HDM control mice. This increase was significantly reduced in NVP-treated mice, reaching basal levels in the NVP 2 weeks group. We did not observe changes in the proportion of neutrophils and lymphocytes between experimental groups. In addition, monocyte presence was reduced in HDM-challenged mice compared to PBS controls (Figure 2A). We next measured serum IgE and IL13 levels and found that both were clearly induced in HDM control mice. Whereas IgE was not affected by NVP, IL13 levels were significantly reduced upon NVP treatment, reaching basal levels in the NVP 2 weeks group (Figure 2B).

Bone marrow cellularity was also assessed in all experimental groups. A significant increase in total cell numbers, eosinophil and neutrophil counts was observed in HDM controls. Interestingly, eosinophil numbers returned to basal levels after one week of NVP treatment, and neutrophil counts were only normalized in the NVP 2 weeks group (Figure 2C). 

### 3.3. Pharmacological Targeting of IGF1R Ameliorates Pulmonary Pathology upon HDM Exposure

**Figure 3:**
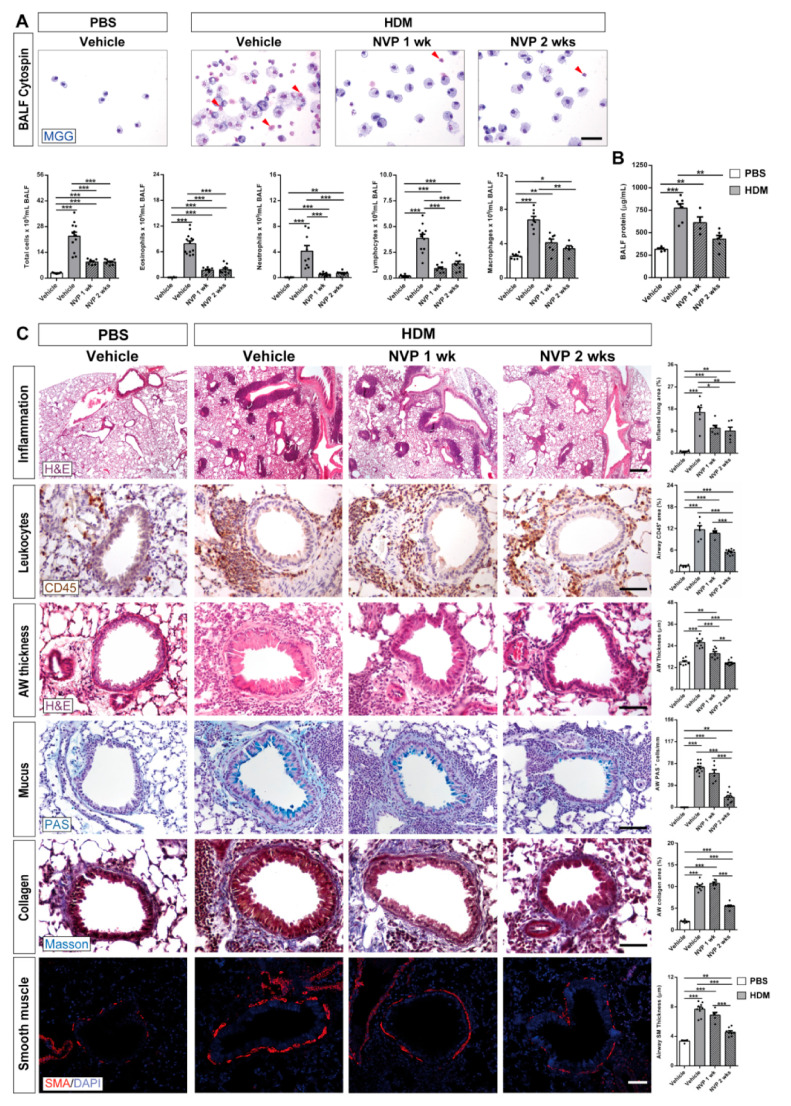
Pharmacological blockade of IGF1R attenuates pulmonary pathology after HDM induced allergy. (**A**) Representative images showing May-Grünwald/Giemsa (MGG) stained BALF cytospin preparations (red arrowheads indicate eosinophils), and total and differential BALF cell counts for eosinophils, neutrophils, lymphocytes and macrophages in HDM-challenged mice treated with NVP vs. controls (*n* = 7–12 mice per group; scale bar: 50 µm). (**B**) Total protein concentration in BALF of HDM-challenged mice treated with NVP vs. controls (*n* = 5–8 mice per group). (**C**) Representative images of lung inflammation and histopathology of the proximal airways, and respective quantifications of inflamed lung areas (%) (H&E), presence of peribronchiolar CD45^+^ area (leukocytes) (%) (brown), airway (AW) epithelium thickness (H&E), number of airway PAS+ cells (mucus-producing cells) (blue), peribronchiolar airway collagen content (%) (Masson in blue) and airway smooth muscle (SM) thickness (SMA in red). These parameters were measured in lung sections from HDM-challenged mice treated with NVP vs. controls (*n* = 6–10 mice per group; scale bars: 50 µm except for the inflammation panel (400 µm)). Quantifications were performed in five different fields in a random way. Data are expressed as mean ± SEM. * *p* < 0.05; ** *p* < 0.01; *** *p* < 0.001 (Mann–Whitney U test or Student´s *t*-test for comparing two groups and Kruskal–Wallis test or ANOVA multiple comparison test for grouped or multivariate analysis).

First, cellularity and total protein levels in bronchoalveolar lavage fluid (BALF) were assessed (Figure 3A,B). Total and differential BALF cells counts were found increased in HDM control mice and this increment was strongly reduced in NVP-treated mice (Figure 3A). In addition, the increase in total protein content in BALF of HDM control mice remained comparable to unchallenged controls in the NVP 2 weeks group (Figure 3B).

Next, we evaluated several airway remodeling indicators including inflamed lung area, leukocyte presence, airway thickness, mucus-producing cells, collagen deposition and smooth muscle (SM) thickness (Figure 3C). Notably, the highly increased values found for all these parameters in HDM-challenged mice were significantly reduced in the NVP 2 weeks group. NVP treatment for one week was less effective, counteracting only inflamed lung area and airway thickness (Figure 3C). 

### 3.4. Pharmacological Blockade of IGF1R Attenuates AHR and Ameliorates Surfactant Deregulation after HDM Challenge

**Figure 4:**
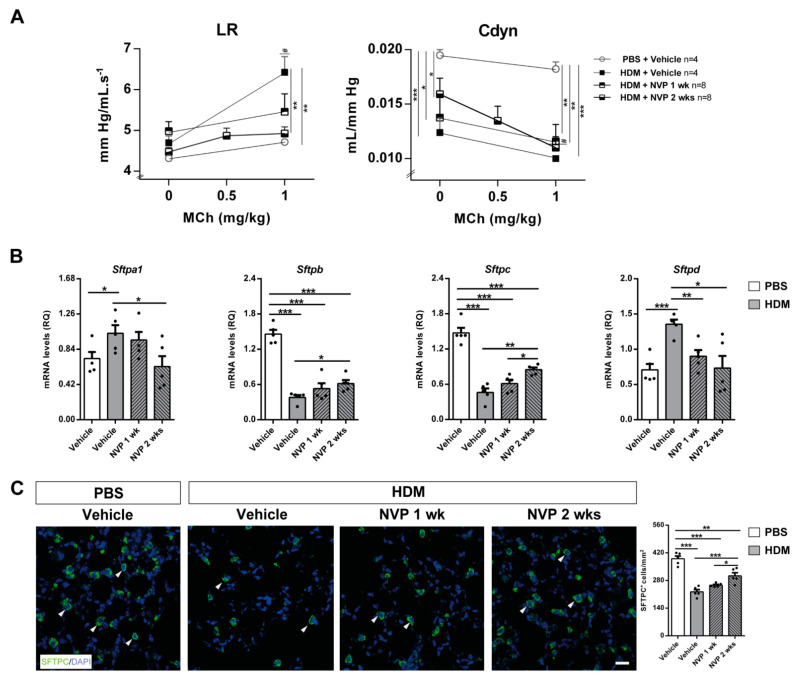
Therapeutic inhibition of IGF1R attenuates AHR and normalizes pulmonary surfactant expression upon HDM-induced allergy. (**A**) Quantification of lung resistance (LR) and dynamic compliance (Cdyn) to methacholine (MCh) evaluated by plethysmography (*n* = 4–8 mice per group) and (**B**) changes in lung tissue mRNA expression surfactant (*Sftp*) markers *Sftpa1, b, c* and *d*, normalized to 18S expression in HDM-challenged mice treated with NVP vs. controls (*n* = 5 mice per group). (**C**) Representative immunostains for SFTPC (green) (white arrowheads), and quantification of the number of SFTPC^+^ cells per unit area (mm^2^) in lung sections from HDM-challenged mice treated with NVP vs. controls (*n* = 5–10 mice per group; scale bar: 50 µm). Data are expressed as mean ± SEM. * *p* < 0.05; ** *p* < 0.01; *** *p* < 0.001; *# p* < 0.05 (comparisons within the same group) (Mann–Whitney U test or Student´s *t*-test for comparing two groups and Kruskal–Wallis test or ANOVA multiple comparison test for grouped or multivariate analysis).

In order to evaluate lung function following HDM-induced allergy, we assessed AHR to methacholine by plethysmography. Methacholine administration induced a marked AHR with increased lung resistance (LR) in HDM controls with respect to PBS challenged mice, whilst mice treated for two weeks with NVP did not show such an increase. However, the dynamic compliance (Cdyn) was reduced in HDM-challenged mice when compared to PBS controls and NVP-treated mice showed a lower reduction in Cdyn (Figure 4A).

Gene expression of the surfactant (*Sftp*) markers a1, b, c and d was evaluated to elucidate how the pharmacological blockade of IGF1R modulates their production. Whereas *Sftpa1* and *Sftpd* mRNA expression levels were significantly increased in HDM controls, *Sftpb* and *Sftpc* levels were severely depleted. Interestingly, NVP treatment reversed these changes, especially in the NVP 2 weeks group, in which *Sftpa1* and *Sftpd* mRNA levels were normalized (Figure 4B). In accordance, immunofluorescence for SFTPC showed that the number of SFTPC^+^ cells was strongly reduced in HDM control mice, and this decrease was counteracted only in the NVP 2 weeks group (Figure 4C). 

### 3.5. Therapeutic Inhibition of IGF1R Halts Expression of Allergic Airway Inflammation Markers after HDM Exposure

**Figure 5:**
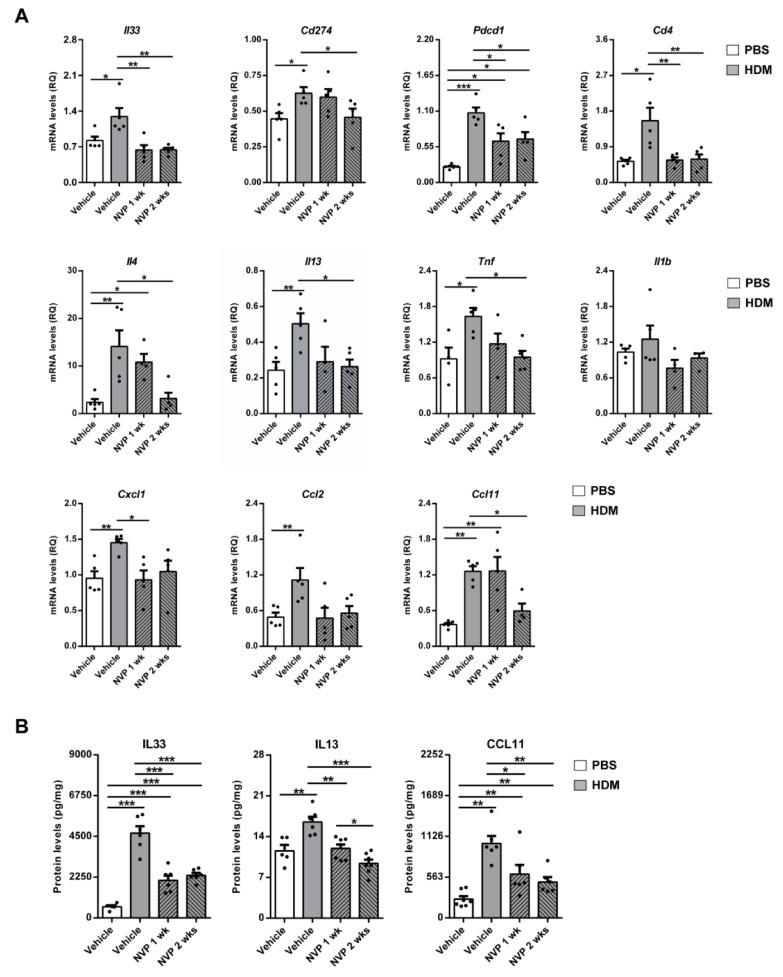
Therapeutic inhibition of IGF1R diminishes expression of allergic airway inflammation markers after HDM exposure. (**A**) Lung tissue mRNA expression levels of *Il33* (dendritic cell activation), *Cd274* (PD-L1) and *Pdcd1* (PD-1) (T cell response), *Cd4* (T cell marker), *Il4* and *Il13* (Th2 cytokines), *Tnf* and *Il1b* (Th1 cytokines), *Cxcl1* (neutrophil chemotaxis), *Ccl2* (macrophage chemotaxis) and *Ccl11* (eosinophil chemotaxis) normalized to 18S expression in HDM-challenged mice treated with NVP vs. controls (*n* = 5 mice per group). (**B**) IL33, IL13 and CCL11 protein levels in lung homogenates from HDM-challenged mice treated with NVP vs. controls (*n* = 5–7 mice per group). Data are expressed as mean ± SEM. * *p* < 0.05; ** *p* < 0.01; *** *p* < 0.001 (Mann–Whitney U test or Student´s *t*-test for comparing two groups and Kruskal–Wallis test or ANOVA multiple comparison test for grouped or multivariate analysis).

Total lung mRNA expression and protein levels of allergic airway inflammation markers were assessed on lung homogenates of HDM-challenged mice treated with NVP vs. controls by qPCR and ELISA, respectively. With the exception of *Il1b*, which did not show any significant difference between groups, mRNA levels of all these markers were strongly induced by HDM (Figure 5A). Whereas *Il33*, *Cd274* (PDL-1), *Cd4*, *Il13*, *Tnf*, *Cxcl1* and *Ccl2* mean levels remained around normal after treatment with NVP, *Il4* and *Ccl11* required NVP treatment for two weeks to amend the HDM response. *Pdcd1* (PD-1) showed a compellingly reduced level of mRNA in both NVP-treated groups (Figure 5A). In agreement, IL33, IL13 and CCL11 protein levels in lung homogenates were clearly induced in HDM controls and significantly reduced in NVP-treated mice (Figure 5B). 

### 3.6. IGF1R Blockade Depleted Bronchiolar Epithelial Differentiation and Goblet Cell Hyperplasia upon HDM-Induced Allergy

**Figure 6:**
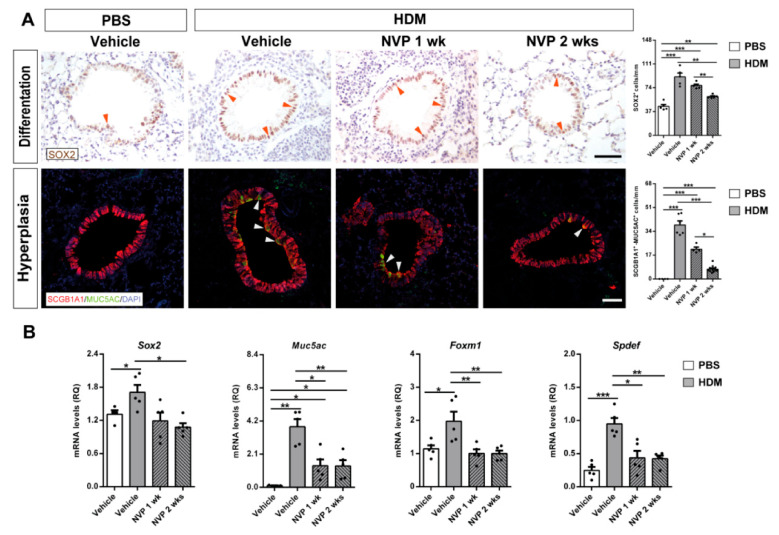
Pharmacological targeting of IGF1R attenuates bronchial differentiation and goblet cell hyperplasia upon HDM-induced allergy. (**A**) Representative immunostains of proximal airways for SOX2 (bronchial differentiation) (brown; orange arrowheads indicate SOX2^+^ cells), as well as double immunofluorescent stains for SCGB1A1 (red) (club cell marker) and MUC5AC (green) (goblet cell hyperplasia) (white arrowheads indicate double SCGB1A1^+^-MUC5AC^+^ cells). Quantification of SOX2^+^ and double SCGB1A1^+^-MUC5AC^+^ cells per epithelium length (mm) in lung sections from HDM-challenged mice treated with NVP vs. controls (*n* = 5–10 mice per group; scale bars: 50 µm). (**B**) Lung mRNA expression levels of *Sox2* (bronchial differentiation) and *Foxm1, Spdef* and *Muc5ac* markers (goblet cell hyperplasia) normalized to 18S expression in HDM-challenged mice treated with NVP vs. controls (*n* = 5 mice per group). Quantifications in lung sections were performed in 5 different bronchi in a random manner. Data are expressed as mean ± SEM. * *p* < 0.05; ** *p* < 0.01; *** *p* < 0.001 (Mann–Whitney U test or Student´s *t*-test for comparing two groups and Kruskal–Wallis test or ANOVA multiple comparison test for grouped or multivariate analysis).

In order to evaluate bronchiolar differentiation and goblet cell hyperplasia, we immunostained SOX2 and MUC5AC, respectively. We observed an increased proportion of SOX2^+^ cells and double stained SCGB1A1^+^-MUC5AC^+^ cells upon HDM challenge, which were significantly reduced in the NVP 2 weeks group (Figure 6A). To complement these data, we also assessed mRNA expression levels of the goblet cell hyperplasia markers *Sox2*, *Muc5ac*, *Foxm1* and *Spdef*. Results on *Sox2* and *Muc5ac* mirror immunostaining data. *Foxm1* and *Spdef* followed mRNA expression profiles of allergic airway inflammation markers. In all cases, IGF1R inhibition with NVP was able to reverse the increase in mRNA expression triggered by the HDM challenge (Figure 6B). 

## 4. Discussion

We aimed to determine if therapeutic targeting of IGF1R ameliorates established allergic airway inflammation in a murine model of HDM-induced asthma. 

HDM-induced allergy has been successfully proven for the study of asthma pathobiology [15,16,24]. Therefore, we deemed that our asthma model was appropriate for testing the in vivo anti-asthmatic efficacy of IGF1R inhibition using the IGF1R TKI inhibitor NVP-ADW742 (NVP). NVP was reported to have an inhibitory effect against IGF1R that was >16-fold more potent than towards InsR, the kinase with the highest homology to IGF1R, and exhibited no symptoms of toxicity [20]. Even though IGF1R activation levels significantly increased in HDM-exposed mice in serum and lung homogenates, NVP treatment counteracted IGF1R phosphorylation in both compartments. We also assessed expression of p-ERK1/2, which is a major IGF1R MAP kinase signaling mediator [7]. In this regard, increased expression of p-ERK1/2 after HDM-induced allergy was attenuated upon treatment with the IGF1R TKI inhibitor NVP. Accordingly, ERK1/2 was reported to stimulate eosinophil chemotaxis, differentiation, cytokine production as well as eotaxin-induced degranulation [25,26,27]. Furthermore, Bates et al. reported that human airway eosinophils respond to several allergic airway inflammation-related chemoattractants with increased activation of the Ras–ERK cascade [28]. 

We have previously shown that mRNA expression of the IGF system genes *Insr*, *Igfbp3* and *Igfbp5* was repressed upon HDM exposure [15]. In particular, increased expression of *Igfbp3* in the NVP 2 weeks group corresponds with its protective role in asthma [14]. In contrast, *Igfbp2* and *Igfbp4* expression depends solely on IGF1R activation since they were only found diminished by NVP treatment. Increased expression of *Igf1* by HDM, which lowered to basal levels upon treatment with NVP, is in accordance with our previous findings [16]. HDM-mediated induction of *Igf1* could be responsible for IGF1R phosphorylation to promote the asthmatic response, and therefore supports why targeting IGF1R with NVP ameliorates HDM-induced asthma. Accordingly, IGF1 was reported to be involved in airway inflammation and remodeling in asthma and increased in serum of asthmatic patients [7,13,14,29].

On the whole, two weeks of NVP treatment were required to ameliorate the hallmarks of established HDM-induced allergy including AHR and airway remodeling. Notably, many of the typical inflammatory allergic features such as eosinophilia, increased cytokine levels and inflamed lung areas were already normalized after one week of NVP treatment. It should be emphasized that in our mouse model of asthma, the allergic phenotype was reported to be present two weeks after HDM exposure [16].

Lung function was improved upon treatment with NVP, which also normalized pulmonary surfactant expression. All these peculiarities were previously reported in *Igf1r*-deficient mice [15]. The fact that AHR is considered to be dependent on airway remodeling [30,31] is consistent with ameliorated remodeling features upon NVP treatment. Pulmonary surfactant proteins play an essential role in lung function and homeostasis [32]. Whereas SFTPB and SFTPC showed a critical role in the preservation of lung function, SFTPA and SFTPD demonstrated immunomodulatory roles during allergic airway inflammation [33,34,35,36]. According to recovered density of SFTPC^+^ alveolar type II cells following NVP treatment, alveolar type II cells were shown to be major contributors to surfactant synthesis [37].

Lung inflammation in allergic asthma is orchestrated by activation of CD4^+^ T lymphocytes to stimulate the release of inflammatory mediators and elicit eosinophilia [38,39]. Accordingly, HDM exposure caused increased leukocytosis in the bone marrow, blood and BALF, mainly due to eosinophils, and elevated serum IgE and IL13 levels, as we reported [15,16]. Of relevance, NVP treatment ameliorated all these features. NVP treatment also decreased the expression of the T cell-related genes *Cd274* (PDL-1), *Pdcd1* (PD-1), *Tnf* and *Cd4*. *Cd274* and *Pdcd1* were reported to be important for the activation of T lymphocytes in asthma and PD-1 expression increased in T-CD4^+^ lymphocytes of asthmatic patients [40,41,42]. Moreover, TNF was reported to be required for allergen-specific Th2 cell activation and for the development of AHR [43].

NVP treatment also diminished IL13, IL33 and CCL11 levels, reported to be highly induced upon HDM exposure [15,16]. IL33 was reported to be a central activator of dendritic cells during HDM allergic sensitization and to exacerbate allergic bronchoconstriction [44,45,46]. IL13 is a central mediator of allergic asthma and its blockade in mice reduces eosinophilia and airway remodeling in response to HDM [47]. CCL11 was reported to be released by bronchial epithelial cells in response to cytokines such as IL4, IL13 and TNF, and is essential for lung eosinophilia and AHR development [48,49,50]. NVP treatment also reduced the presence of CD45^+^ leucocytes in the lung, which were shown to be involved in allergic airway inflammation [51,52]. 

NVP treatment attenuated bronchial epithelial differentiation and goblet cell hyperplasia. We previously reported that IGF1R is required for proper club cell differentiation in mice [53]. Accordingly, club cells were reported to play a major role in bronchial asthma and SOX2 is required for goblet cell differentiation after allergen sensitization [49,54]. Upon allergen stimulation, FOXM1 induces differentiation of club cells into goblet cells through transcriptional activation of SPDEF. Then, increased MUC5AC expression by SPDEF in goblet cells contributes to mucus hyperproduction and AHR [31,55,56]. 

## 5. Conclusions

Our results demonstrate that the pharmacological blockade of IGF1R with NVP-ADW742 ameliorates HDM-induced allergy, and places IGF1R as a potential pharmacological target for future therapeutic approaches in asthma. In addition, IGF1R could be considered a promising candidate biomarker in asthma. 

## Data Availability

The data that support the findings of this study are available from the corresponding author upon reasonable request.

## Supplementary Materials

The following are available online at https://www.mdpi.com/2227-9059/9/8/912/s1. Figure S1: Follow-up of the body weight gain upon treatment with the IGF1R inhibitor NVP-ADW742, Figure S2: Treatment with DMSO does not induce inflammation in the lungs of C57BL/6J mice, Table S1: Primer sets used for qPCR.
