# Correction: Alfaro-Arnedo et al. IGF1R as a Potential Pharmacological Target in Allergic Asthma. *Biomedicines* 2021, *9*, 912

**DOI:** 10.3390/biomedicines10040733

**Published:** 2022-03-22

**Authors:** Elvira Alfaro-Arnedo, Icíar P. López, Sergio Piñeiro-Hermida, Álvaro C. Ucero, Francisco J. González-Barcala, Francisco J. Salgado, José G. Pichel

**Affiliations:** 1 Lung Cancer and Respiratory Diseases Unit, Center for Biomedical Research of La Rioja (CIBIR), Fundación Rioja Salud, 26006 Logroño, Spain; ealfaro@riojasalud.es (E.A.-A.); iplgarcia@riojasalud.es (I.P.L.); 2 Telomeres and Telomerase Group, Molecular Oncology Program, Spanish National Cancer Centre (CNIO), 28029 Madrid, Spain; spineiro@cnio.es; 3 Thoracic Oncology, Research Institute Hospital 12 de Octubre, 28041 Madrid, Spain; acuceroh@ucm.es; 4 Department of Physiology, Faculty of Medicine, Complutense University, 28040 Madrid, Spain; 5 Department of Respiratory Medicine, University Hospital of Santiago de Compostela (CHUS), 15706 Santiago de Compostela, Spain; francisco.javier.gonzalez.barcala@sergas.es; 6 Health Research Institute of Santiago de Compostela (FIDIS), 15706 Santiago de Compostela, Spain; 7 Spanish Biomedical Research Networking Centre-CIBERES, 15706 Santiago de Compostela, Spain; 8 Department of Biochemistry and Molecular Biology, Faculty of Biology-Biological Research Centre (CIBUS), Universidad de Santiago de Compostela, 15706 Santiago de Compostela, Spain; franciscojavier.salgado@usc.es

In the original article [1], there was a mistake in Figure 4 as published. We made a mistake when choosing the representative image for the second panel in Figure 4C (corresponding to the “HDM +Vehicle” treatment). The corrected Figure 4 appears below. 

**Figure 4:**
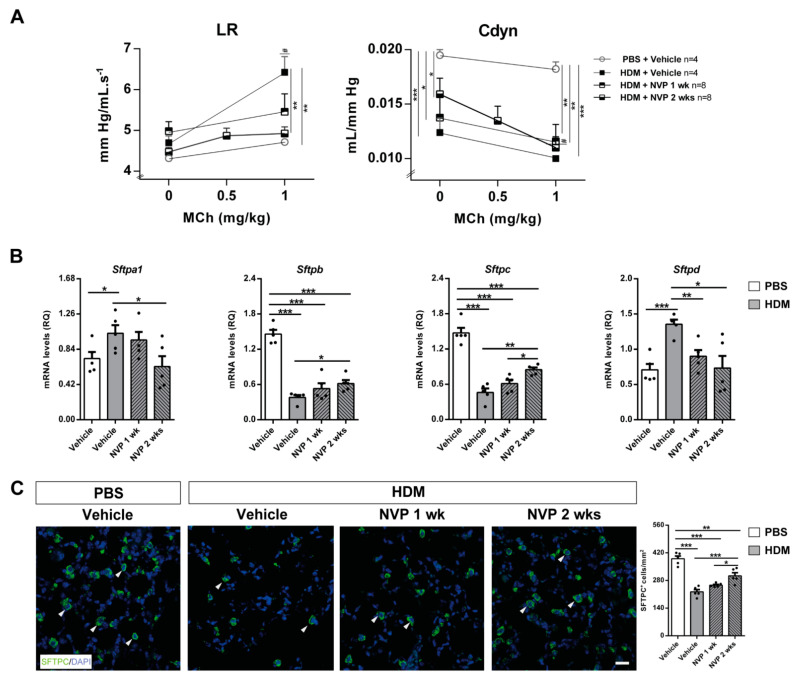
Therapeutic inhibition of IGF1R attenuates AHR and normalizes pulmonary surfactant expression upon HDM-induced allergy. (**A**) Quantification of lung resistance (LR) and dynamic compliance (Cdyn) to methacholine (MCh) evaluated by plethysmography (*n* = 4–8 mice per group) and (**B**) changes in lung tissue mRNA expression surfactant (*Sftp*) markers *Sftpa1*, *b* *c*, and *d*, normalized to 18S expression in HDM-challenged mice treated with NVP vs. controls (*n* = 5 mice per group). (**C**) Representative immunostains for SFTPC (green) (white arrowheads), and quantification of the number of SFTPC^+^ cells per unit area (mm^2^) in lung sections from HDM-challenged mice treated with NVP vs. controls (*n* = 5–10 mice per group; scale bar: 50 µm). Data are expressed as mean ± SEM. * *p* < 0.05; ** *p* < 0.01; *** *p* < 0.001; *# p* < 0.05 (comparisons within the same group) (Mann–Whitney U test or Student’s *t*-test for comparing two groups and Kruskal–Wallis test or ANOVA multiple comparison test for grouped or multivariate analysis).

The authors apologize for any inconvenience caused and state that the scientific conclusions are unaffected. The original publication has also been updated.
