# Supplementary material for: IGF1R as a Potential Pharmacological Target in Allergic Asthma
Source: Biomedicines. 2021 Jul 29;9(8):912. doi: 10.3390/biomedicines9080912 (PMC8389607; doi:10.3390/biomedicines9080912)
Supplement: Supplementary file 1 [file biomedicines-09-00912-s001.zip › biomedicines-1314799-supplementary.pdf]

Supporting information for

IGF1R as a potential pharmacological target in allergic asthma

Elvira Alfaro-Arnedo, Icíar P. López, Sergio Piñeiro-Hermida, Álvaro C. Ucero, Francisco J. González-Barcala, Francisco J. Salgado and José G. Pichel*

Files included:

Figure S1. Follow-up of the body weight gain upon treatment with the IGF1R inhibitor NVP-ADW742.

Figure S2. Treatment with DMSO does not induce inflammation in the lungs of C57BL/6J mice.

TableS1. Primer sets used for qPCR.

Supplementary Methods.

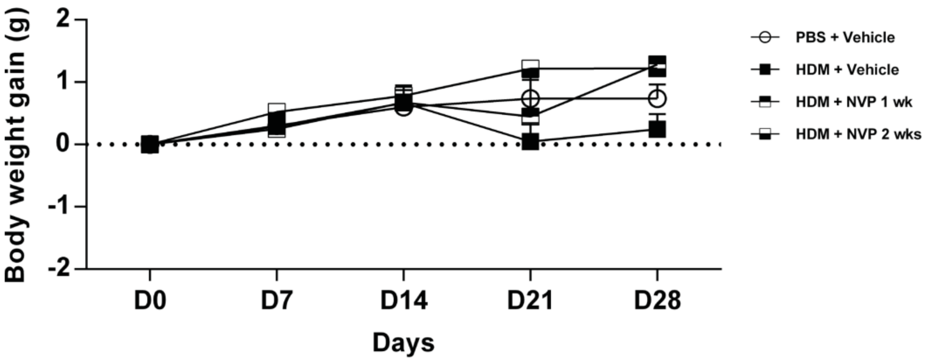

Figure S1. Follow-up of the body weight gain upon treatment with the IGF1R inhibitor NVP-ADW742. Weekly follow-up of the body weight gain in HDM-challenged mice treated with the TKI NVP-ADW742 vs. controls (n = 5 mice per group). Data are expressed as mean ± SEM. (Mann-Whitney U test or Student´s t-test for comparing 2 groups and Kruskal-Wallis test or ANOVA multiple comparison test for grouped or multivariate analysis).

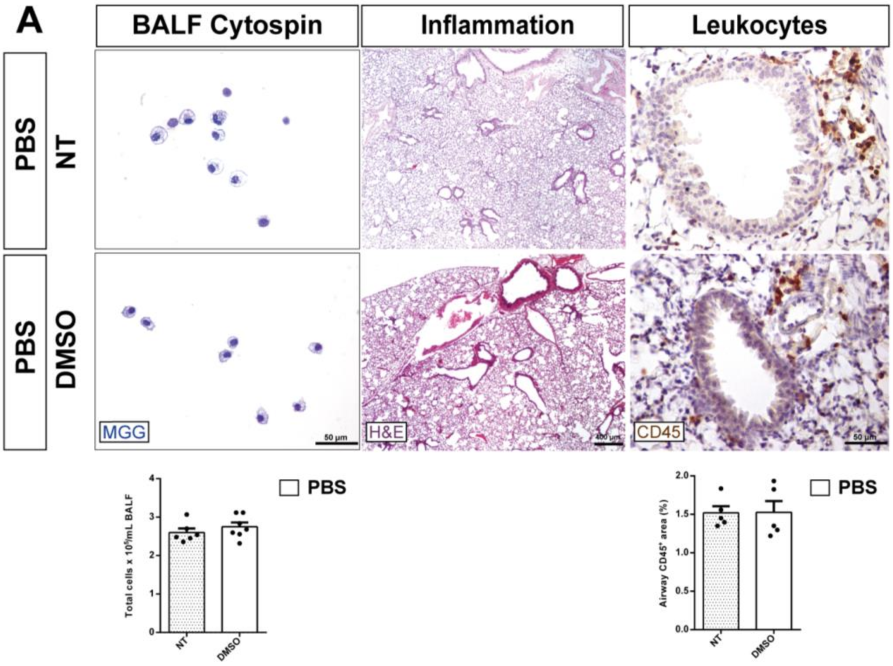

Figure S2. Treatment with DMSO does not induce inflammation in the lungs of C57BL/6J mice. (**A**) Representative BALF cytospin preparations (May-Grünwald Giemsa (MGG) (left), and images of proximal airways showing H&E (center) and CD45 (brown) stainings (right) and quantification of total BALF cell counts, and CD45^{+} (leukocytes) area (%) in lung sections from C57BL/6J mice treated with 2% DMSO vs. untreated controls (n = 5-7 mice per group). Data are expressed as mean ± SEM. (Student´s t-test for comparing 2 groups).

TableS1. Primer sets used for qPCR.

Gene	Accession No.	Forward primer (5´-3´)	Reverse primer (5´-3´)
Ccl2	NM_011333.3	CACCAGCCAACTCTCACTGA	CGTTAACTGCATCTGGCTGA
Ccl11	NM_011330.3	GAGAGCCTACAGAGCCCAGA	ACCGTGAGCAGCAGGAATAG
Cd4	NM_013488.2	ATGTGGAAGGCAGAGAAGGA	TGGGGTATCTTGAGGGTGAG
Cd274	NM_021893.3	CATACCGCAAAATCAACCAG	CACTTCTCTTCCCACTCACG
Cxcl1	NM_008176.3	ATCCAGAGCTTGAAGGTGTTG	GTCTGTCTTCTTTCTCCGTTACTT
Foxm1	NM_008021.4	CCTGCTTACTGCCCTTTCCT	CACACCCATCTCCCTACACC
Igf1	NM_010512	CAGAAGCGATGGGGAAAAT	GTGAAGGTGAGCAAGCAGAG
Igfbp2	NM_008342	GGGAGTGCTGGTGTGTGA	CTGCTGGTGTTCGGGATG
Igfbp3	NM_008343.2	GCCCTCTGCCTTCTTGATTT	TCACTCGGTTATGGGTTTCC
Igfbp4	NM_010517.3	TGTGAGATTGGATTGTGTGTGT	TAGAGATGGCGGGATAGGAG
Igfbp5	NM_010518.2	GATGAGACAGGAATCCGAACAAG	AATCCT TTGCGGTCACAGTTG
Igfbp6	NM_008344	AGGAGAGCAAACCCCAAGGA	TGAACAGGATTGGGCCGTATA
Il1b	NM_008361.3	GCAACTGTTCCTGAACTCAACT	ATCTTTTGGGGTCCGTCAACT
Il4	NM_021283.2	CCTCACAGCAACGAAGAACA	CGAAAAGCCCGAAAGAGTC
Il13	NM_008355.3	GCCTCCCCGATACCAAAAT	CTTCCTCCTCAACCCTCCTC
Il33	NM_133775.2	GCCTTGCTCTTTCCTTTTCTC	TCGGTTGTTTTCTTGTTTTGC
Insr	NM_010568.2	TCCTGAAGGAGCTGGAGGAGT	CTTTCGGGATGGCCTGG
Muc5ac	NM_010844.1	CACACACAACCACTCAACCA	TCTCTCTCCGCTCCTCTCAA
Pdcd1	NM_008798.2	TCAAGGCATGGTCATTGGTA	GCTCCTCCTTCAGAGTGTCG
Rn18s	NR_003278.3	ATGCTCTTAGCTGAGTGTCCCG	ATTCCTAGCTGCGGTATCCAGG
Sftpa1	NM_023134.4	CCATCGCAAGCATTACAAAG	CACAGAAGCCCCATCCAG
Sftpb	NM_147779.2	CTGCTGCTTCCTACCCTCTG	ATCCTCACACTCTTGGCACA
Sftpc	NM_011359	GAAGATGGCTCCAGAGAGCATC	GGACTCGGAACCAGTATCATGC
Sftpd	NM_009160.2	TGAGAATGCTGCCATACAGC	GAATAGACCAGGGGCTCTCC
Sox2	U_31967.1	AACCAAGACGCTCATGAAGAAG	CTGCGAGTAGGACATGCTGTAG
Spdef	NM_013891.4	GGCCAGCCATGAACTATGAT	GGTAGACAAGGCGCTGAGAG
Tnf	NM_013693.3	GCCTCTTCTCATTCCTGCTTG	CTGATGAGAGGGAGGCCATT

Supplementary Methods

HDM Sensitization Protocol and Therapeutic Inhibition of IGF1R

Inbred C57BL/6J female mice were purchased from Charles River Laboratories, Inc., and maintained under specific pathogen-free (SPF) conditions in laminar flow caging at the CIBIR animal facility. All experiments and animal procedures conducted were carried out following the guidelines of the European Communities Council Directive (86/609/EEC), and were revised and approved by the CEAA/CIBIR (Gobierno de La Rioja) Bioethics Committee (ref. JGP01_v2). C57BL/6J mice were intranasally challenged either with 40 µg of house dust mite (HDM) extract (Greer Laboratories Inc, Lenoir, NC) in 20 µL PBS (2 mg/mL) or an equal volume of PBS under light isoflurane anesthesia five days a week for four weeks. We generated four experimental groups: PBS + vehicle (PBS i.n. + 2% DMSO in saline i.p. twice daily between D14 and D27), HDM + vehicle (HDM i.n. + 2% DMSO i.p. twice daily between D14 and D27), HDM + NVP-ADW742 1 wk (Selleckchem, Houston, TX) (HDM i.n + 10 mg/kg of NVP-ADW742 in 2% DMSO i.p. twice daily between D21 and D27) and HDM + NVP-ADW742 2 wks (HDM i.n. + 10 mg/kg of NVP-ADW742 in 2% DMSO i.p. twice daily between D14 and D27) (Fig 1a). To evaluate the effect of DMSO on lung inflammation, we included an additional control group of mice, which was compared with the group PBS + DMSO (PBS i.n. + 2% DMSO in saline i.p. twice daily between D14 and D27). To generate the additional control group, inbred C57BL/6J mice were intranasally challenged with PBS five days a week for four weeks. Mice were weighed on days (D) D0, D7, D14, D21, D28 of the protocol to assess body

weight gain. Females were used due to their reported higher susceptibility to allergic airway inflammation [1].

In Vivo Assessment of Lung Function

In vivo measurement of lung function was performed 24h after the last HDM exposure (D28). Mice were anesthetized by intraperitoneal injection (2.5 µL/g) with the anaesthetic combination of fentanyl citrate (Fentanest 0.05 mg/mL, KERN PHARMA), medetomidine hydrochloride (Domtor 1 mg/mL, ECUPHAR) and midazolam hydrochloride (Midazolam Accord 5 mg/mL, ACCORD) and intubated with a 24-gauge catheter (BD, Franklin lakes, NJ). Mice received a single intravenous tail injection of 1 mg/kg of methacholine (MCh) (Sigma-Aldrich, St. Louis, MO) [2] and lung function was assessed within 5 minutes using a plethysmograph (SCIREQ, Montreal, Canada), having previously measured basal lung function. Additionally, 0.5 mg/kg of MCh were administrated to HDM-challenged mice treated with NVP-ADW742 for 2 weeks (HDM + NVP 2 wks). A MiniVent (Harvard Apparatus, Holliston, MA) was connected to the plethysmograph and the tracheal cannula for animal ventilation at 10 mL/kg of tidal volume and 150 breaths per minute. Data were measured by 2 pressure transducers that detect pressure variations in the chamber (flow) and in the tracheal cannula (pressure) [3].

Sample Collection and Preparation

After lung function measurement, mice were euthanized by intraperitoneal injection of 10 µL/g of a ketamine-xylazine anesthetic combination in saline (300:30 mg/kg, respectively). Blood was collected by cardiac puncture, and then 50 µL were mixed with 1 mL of ACK Lysing Buffer (Thermo Fisher

Scientific, Waltham, MA) and centrifuged at 300 xg for 5 min at 4°C after 15 min of incubation. Following aspiration of the supernatants, pellets were washed with 500 μL PBS and centrifuged at 300 xg for 5 min at 4°C, repeating this step once more. The supernatants were discarded and 200 μL of PBS were added to the pellets to prepare the cytospin preparations by centrifugation of the slides at 1500 rpm for 5 min (Cytospin 4, Thermo Fisher Scientific, Waltham, MA). Serum was obtained by centrifugation at 3000 xg for 10 min at 4°C and stored at -80°C until further use. Next, lungs were lavaged twice with 0.8 mL of cold PBS to obtain the bronchoalveolar lavage fluid (BALF) which was centrifuged at 15700 xg for 5 min at 4°C. The BALF supernatants were stored at -80°C to subsequently assess total protein concentration in BALF using the Pierce BCA Protein Assay Kit (Thermo Fisher Scientific). In addition, the BALF pellets were suspended in 500 μL ACK Lysing Buffer (Thermo Fisher Scientific) and centrifuged at 3300 xg for 5 min at 4°C after 10 min of incubation. The supernatants were discarded and 500 μL PBS were added to the pellet to prepare the cytospin preparations by centrifugation of the slides at 1500 rpm for 5 min (Cytospin 4, Thermo Fisher Scientific). Following lung dissection, right lung lobes were separated, snap-frozen in liquid nitrogen and stored at -80°C for quantitative PCR (qPCR) and ELISA analyses, and the left lung lobe was fixed by inflation with 4% formaldehyde for 8-10h and subsequently embedded in paraffin for histopathology and immunohistochemistry [4].

Bone marrow (BM) isolation was carried out following dissection of the femur. After centrifugation at 10000 xg for 15 seconds, BM was suspended in 500 μL PBS and centrifuged at 300 xg for 5 min at 4°C. Following aspiration

of the supernatants, BM pellets were resuspended in 500 µL ACK Lysing Buffer (Thermo Fisher Scientific) and centrifuged at 300 xg for 5 min at 4°C after 10 min of incubation. The supernatants were discarded and 1 mL PBS was added to the pellets to prepare the cytospin preparations by centrifugation of the slides at 1500 rpm for 5 min (Cytospin 4, Thermo Fisher Scientific) [5].

Quantification of Blood, BALF and Bone Marrow

Total cell number was counted and expressed as cells/mL in BALF and BM, and as a percentage in peripheral blood. Differential cell counts were performed on May-Grünwald/Giemsa (Sigma-Aldrich, St. Louis, MO) stained cytospins, counting a minimum of 300 cells per slide in BALF, BM and blood cytospins. Cells were determined to be macrophages, lymphocytes and neutrophils using standard morphology criteria [4,6].

Histopathologicaland Immunostaining Analysis

Paraffin-embedded left lungs were cut into 3 µm sections for histology and immunohistochemistry. Hematoxylin and eosin (H&E) staining was performed for the quantification of inflammation expressed as the percentage (%) of inflamed lung area (accumulation of inflammatory cells defined as darker H&E stained foci) to total section surface, and to asses airway thickness (µm) by means of three different measurements per airway. Periodic acid-Schiff (PAS) staining served to evaluate the number of mucus-producing cells (airway PAS$^+$ cells/mm) and Masson´s trichrome staining was for quantifying collagen deposition (airway collagen area (%). Immunostaining was performed using the following antibodies: p-ERK1/2 (p-42/44) (Clone E10 1:110, Cell signalling

technology), CD45 (Clone D3F8Q 1:900, Cell signaling technology, Danvers, MA), SMA (Clone 1A4 1:400, Sigma-Aldrich, St. Louis, MO), Prosurfactant Protein C (SFTPC) (1:200, EMD Millipore, Burlington, MA), SOX2 (Clone C70B1 1:100, Cell Signaling Technology), CC10 (Clone T18 1:400, Santa Cruz Biotech. Inc., Dallas, TX), and MUC5AC (Clone 45M1 1:50, Thermo Fisher Scientific). p-ERK1/2 (p-42/44), CD45 and SMA antibodies were used to evaluate airway p-ERK1/2$^+$ and CD45$^+$ (leukocytes) areas (%), and airway smooth muscle (SM) thickness (µm) by means of three different measurements per airway. SFTPC antibody was used todetermine the number of SFTPC$^+$ cells per unit area (mm^2) of lung tissue. SOX2, CC10 and MUC5AC antibodies were used to quantify SOX2$^+$ cells/mm of bronchi (differentiation), and for the assessment of goblet cell hyperplasia (SCGB1A1$^+$-MUC5AC$^+$ cells/mm), respectively. Fiji opensource image processing software package v1.48r (http://fiji.sc) was used to quantify inflamed lung and CD45$^+$ (percentage of DAB) areas (%), airway collagen area (%), airway and smooth muscle thickness and epithelium length measurements. Quantifications in lung sections were performed in 5 different bronchi per animal in a random way.

RNA Isolation, Reverse Transcription and qPCR

Inferior right lung lobes were homogenized in TRIzol (Invitrogen, Carlsbad, CA), and RNA was isolated using an RNeasy Mini Kit (Qiagen, Hilden, Germany) and reverse-transcribed to cDNA using SuperScript II First-Strand Synthesis System (Invitrogen). cDNA samples were amplified by qPCR in triplicate reactions for each primer pair assayed on a 7300 Real-Time PCR

Instrument (Applied Biosystems, Foster City, CA), using SYBR Premix Ex Taq (Takara Bio Inc., Kusatsu, Japan) [4].

Mouse ELISAS

Total serum IgE, IL13 and p-IGF1R levels in mouse were assessed with the IgE (Abcam, Cambridge, UK), IL13 Duoset (R&D systems) and PathScanphospho-IGF-I receptor β (Tyr1131) sandwich (Cell Signaling Technology) ELISA kits. IL13, IL33 and CCL11 cytokines, and p-IGF1R levels were determined in mouse lung lysates. Superior right lung lobes were homogenized in RIPA Buffer (Thermo Scientific) containing a protease-phosphatase inhibitor mixture (Roche, Basel, Switzerland), and total protein concentration was determined with the Pierce BCA Protein Assay Kit (Thermo Fisher Scientific). p-IGF1R levels were quantified with the PathScanphospho-IGF-I receptor β (Tyr1131) sandwich ELISA kit (Cell Signaling Technology), and cytokines using mouse IL13 Duoset, and IL33 and CCL11 Quantikine ELISA Kits (R&D systems, Minneapolis, MN) in volumes of lysates normalized to total lung protein levels.

Statistics

Statistical analyses were carried out using SPSS Statistics Software v21 for Windows (IBM, Armonk, NY). For all analysis, a p value <0.05 was considered statistically significant.

References

1. Melgert BN, et al. Female mice are more susceptible to the development of allergic airway inflammation than male mice. *Clin. Exp. Allergy* 2005;35:1496–1503.

2. Zoltowska Nilsson AM, Lei Y, Adner M, Nilsson GP. Mast cell-dependent IL-33/ST2 signaling is protective against the development of airway hyperresponsiveness in a house dust mite mouse model of asthma. *Am. J. Physiol. Lung Cell. Mol. Physio.* 2018;314:L484–L492.

3. Ucero AC et al. Fra-2 – expressing macrophages promote lung fibrosis. *JClin Invest* 2019;129:3293–3309.

4. Piñeiro-Hermida S, Alfaro-Arnedo E, Gregory JA, Torrens R, Ruíz-Martínez C, Adner M, López IP, Pichel JG. Characterization of the acute inflammatory profile and resolution of airway inflammation after Igf1r-gene targeting in a murine model of HDM-induced asthma. *PLoS One* 2017b;12:1–20.

5. Piñeiro-Hermida S, López IP, Alfaro-Arnedo E, Torrens R, Iñiguez M, Alvarez-Erviti L, Ruíz-Martínez C, Pichel JG. IGF1R deficiency attenuates acute inflammatory response in a bleomycin-induced lung injury mouse model. *Sci Rep* 2017c;7:1–13.

6. Piñeiro-Hermida S, Gregory JA, López IP, Torrens R, Ruíz-Martínez C, Adner M, Pichel JG. Attenuated airway hyperresponsiveness and mucus secretion in HDM-exposed Igf1r-deficient mice. *Allergy* 2017a;72:1317–1326.
